# Renal Lymphangiectasia: The Transcending of Serendipity

**DOI:** 10.7759/cureus.56669

**Published:** 2024-03-21

**Authors:** Gina Paola Ricardo Ossio, Diana Marcela Gallo Orjuela, Juanita Salazar Agudelo, Camilo Gonzalez Gomez, Dennys Tenelanda Lopez

**Affiliations:** 1 Medical Affairs, Universidad del Rosario, Bogota, COL; 2 Clinical Research, Universidad Metropolitana de Barranquilla, Barranquilla, COL; 3 Epidemiology and Public Health, International University of La Rioja, Rioja, ESP; 4 Emergency, Clinica CES, Medellin, COL; 5 Emergency, Pablo Tobon Uribe Hospital, Medellin, COL; 6 Dentistry, National University of Chimborazo, Riobamba, ECU

**Keywords:** magnetic resonance imaging, renal lymphangiectasia, lymphatic system, lymphangioma, pocus (point of care ultrasound)

## Abstract

Renal lymphangiectasia, a rare entity of the renal lymphatic system affecting both genders and all ages, can manifest bilaterally or unilaterally and has been referred to by various terms, such as renal lymphangiomatosis, renal lymphangioma, and others. Distinguishing this condition from common pathologies, such as polycystic kidney disease or hydronephrosis, is crucial. This article presents an innovative clinical case of unilateral renal lymphangiectasia in a 67-year-old woman with a relevant medical history. Detection was achieved by ultrasound in primary care using the point-of-care ultrasound (POCUS) technique under the focused assessment with sonography in trauma (FAST) protocol, revealing findings suggestive of renal lymphangiomatosis. This case highlights the utility of advanced technologies, such as bedside ultrasound, in addressing and transforming the approach to rare medical conditions, offering a compelling reminder of the positive influence of technological innovation in clinical practice.

## Introduction

Renal lymphangiectasia is an unusual and benign disorder that affects the lymphatic vessels of the kidney and whose origin is not yet completely known. Some anomalies associated with renal lymphangiectasia include the creation of perinephric arteries and possible mechanisms of abdominal injury that impede renal lymphatic drainage. Furthermore, research in mice reveals a relationship with vascular endothelial growth factor C (VEGF-C), which could be involved in its development [1]. This alteration favors the dilation of the renal lymphatic systems, which gives rise to fluid-filled cavities, generally simple cysts [2].

The symptoms of renal lymphangiectasia are diverse, from the absence of symptoms to hematuria, flank discomfort (common in adults), palpable abdominal mass (more common in children), and even elevated blood pressure [3]. The diagnosis depends mainly on radiological images but can be difficult because of the differential diagnosis with other diseases, such as polycystic kidney disease or hydronephrosis [4]. However, useful tools such as point-of-care ultrasound (POCUS) refer to the usefulness of ultrasound at the patient's bedside. POCUS has become an essential tool in clinical practice, as it allows instant information about the patient's anatomy to be obtained without needing to send the patient to the radiology service in the first place [5]. It is a tool increasingly used in various areas, such as emergencies, critical care, and cardiology, and is especially effective for primary care doctors such as internists [6].

We present an unusual case of unilateral renal lymphangiectasia in a 67-year-old female patient, unexpectedly detected through an ultrasound scan performed in primary care after a fall with blunt trauma to the left hip. It highlights the importance of POCUS for the early detection of renal problems and highlights the relevance of early recognition of renal lymphangiectasia in primary care. This case highlights the crucial role of ultrasound in the follow-up of this rare condition due to its cost-effectiveness and noninvasive characteristics. Therefore, this case is a substantial contribution to knowledge, setting a precedent for future research in the diagnosis and management of renal lymphangiectasia.

## Case presentation

This is a case report about a 67-year-old female patient with a pathological history of hypothyroidism on hormonal replacement, The American College of Radiology Thyroid Imaging Reporting and Data Systems (TI-RADS) 4 thyroid nodule (under follow-up), arterial hypertension, and a surgical history of urethrocystopexy, colpopexy with sacrospinous fixation, and hysterectomy.

She went to a follow-up appointment with internal medicine to monitor her underlying pathologies. However, during the consultation, she reported pain in her left hip after falling from her height with blunt trauma to the hip. Although an initial x-ray of the hip revealed no visible abnormalities, mild discomfort persisted throughout the medical evaluation, with no distinctive physical findings.

**Figure 1:**
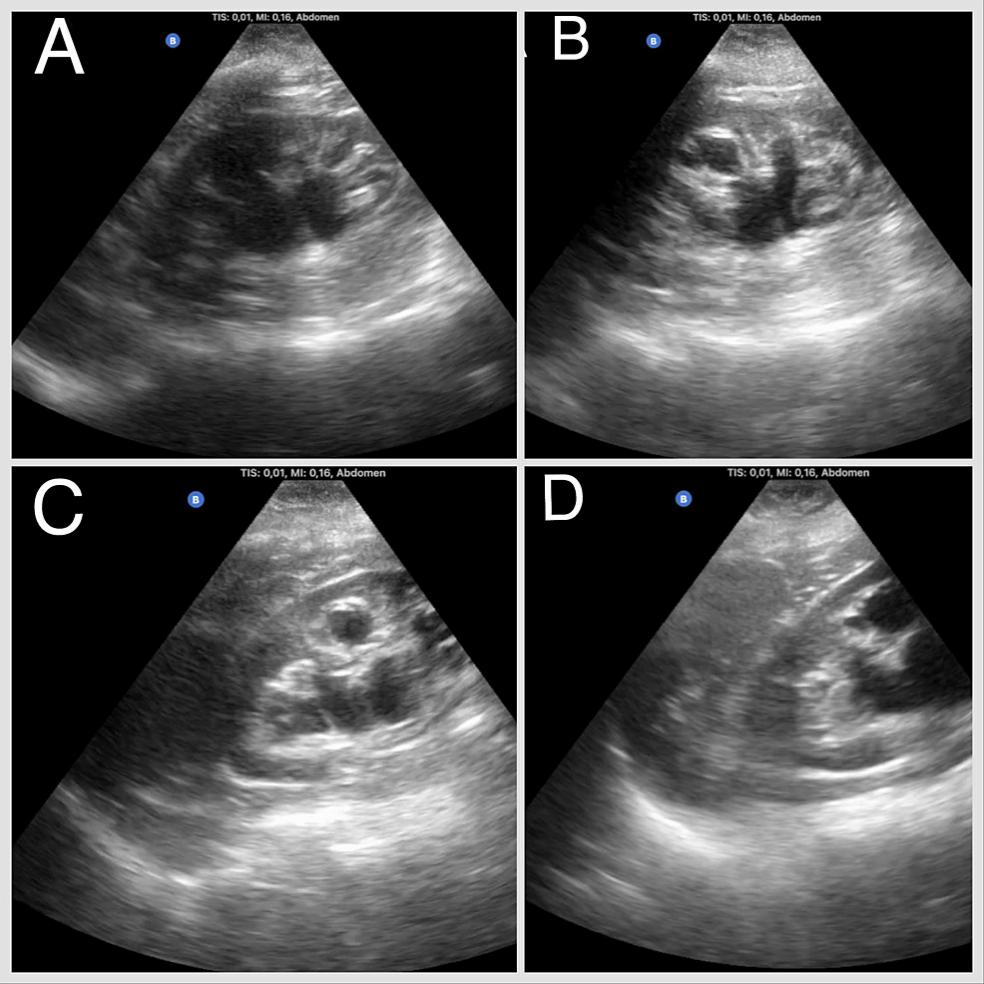
Renal ultrasound: longitudinal plane of the left kidney (A), (B), (C), and (D). Mild-to-moderate pyelocaliectasis of linear appearance without evidence of proximal obstructive factor.

Under the FAST protocol, a bedside ultrasound was performed on the patient, considering POCUS, with no evidence of free fluid in the peritoneal cavity. However, findings in the left kidney, compatible with a mild-to-moderate pyelocaliectasis of linear appearance without evidence of proximal obstruction (Figure 1), made us expand studies to identify a probable etiology. Therefore, urotomography and kidney function tests were requested under normal parameters: creatinine 0.63 mg/dL and creatinine clearance by CKD-EPI of 92.82 mL/min/1.73 m^2^.

**Figure 2:**
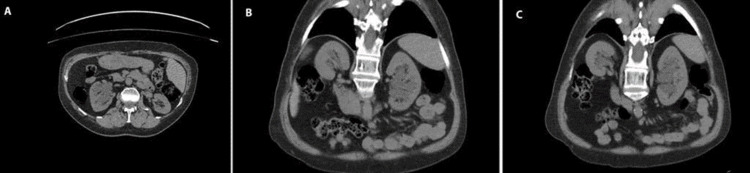
Left renal lymphangiectasia. Urotomography in axial (A) and coronal (B,C) sections shows multiple paraphyletic cystic images located in the renal sinus.

The urotomography in the left kidney identifies multiple paraphyletic cystic images. These are located in the renal sinus without communication between them or apparent dilation of the pyeloinfundibular collecting cavities, which may correspond to cystic lymphangiectasia of the renal sinus (Figure 2).

**Figure 3:**
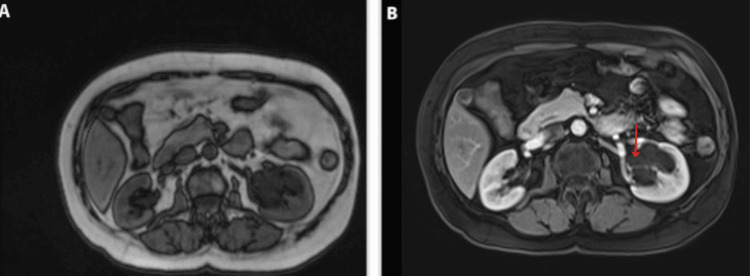
Left renal lymphangiectasia. MRI cystic image without left renal paracalyceal enhancement measuring 40x40x60 mm (transverse, cephalocaudal app). Axial section T1F (A), Axial T1 contrast-enhanced slice (B). Evidence of the pyelocaliceal system during the removal of contrast medium from the visualized cystic lesion, with no findings of extrinsic compression of the pyelocaliceal system.

In connection with investigations of the extent of renal architecture and diagnostic confirmation, abdominal magnetic resonance imaging (MRI) was performed, both plain and contrast-enhanced (Figure 3). The findings indicated kidneys of ordinary size with maintained corticomedullary differentiation but with compression of the renal parenchyma, giving a thinner-than-normal appearance. Additionally, a 5 mm non-enhancing cyst was discovered in the left kidney, and a non-enhancing cystic development in the left renal paracalyceal region, with dimensions of 40x40x60 mm in the anteroposterior, transverse, and cephalocaudal projections, with evidence of the pyelocaliceal system during the removal of contrast medium from the visualized cystic lesion, with no findings of extrinsic compression of the pyelocaliceal system. These results corroborated the diagnosis of left renal lymphangiectasia.

Although the diagnosis was obtained without signs of decreased renal function, it was decided to monitor the patient carefully in conjunction with the urology service.

## Discussion

Renal lymphangiectasia is a rare and benign malformation of the lymphatic vessels of the kidney, with an etiology that is not entirely understood [7]. However, some of the hypotheses are related to the alteration of the formation of the perinephric vessels, which connect with drainage. The renal lymphatic system has also been proposed as a possible consequence of abdominal trauma leading to retroperitoneal obstruction that compromises lymphatic drainage with subsequent dilation of the lymphatic ducts [8].

Recently, in studies with mice, it is believed that it may be related to a protective response under multiple kidney injuries, minimizing damage and progression to interstitial fibrosis. These effects seem to be associated with an increase in VEGF-C. The mouse model showed a severe cystic kidney phenotype similar to renal lymphangiectasia [1]. Finally, this alteration dilates the renal lymphatic structures, leading to fluid-filled cavities, which are primarily simple cystic spaces, but they may be septated and located mainly in the renal sinus [2].

The symptoms of renal lymphangiectasia can be very diverse, from asymptomatic patients to others with hematuria, flank pain (most common symptom in adults), palpable abdominal mass (most common symptom in children), and even high blood pressure with or without acute kidney injury because of creatinine elevation [3,9].

Radiological identification of renal lymphangiectasia is often challenging by conventional ultrasound as the images generally present as cystic lesions with smooth surfaces and may present internal divisions (septa) that do not communicate with each other. These lesions can show variations in their shape, but they do not offer a solid component inside, which is associated with an increase in cortical echogenicity and a loss of corticomedullary differentiation [10,11]. Based on the abovementioned, ultrasound is a helpful tool for initial suspicion. Still, it has limitations when confirming the diagnosis as differentiating these findings from polycystic kidney disease, hydronephrosis, or a urinoma can be a challenge [4].

The usefulness of ultrasound at the patient's bedside was a fundamental tool for the diagnostic approach and early detection of this finding after trauma [12].

However, other pathologies such as polycystic kidney disease, urinoma, renal lymphoma with perinephric involvement, nephroblastomatosis, and other causes involving multiple renal masses, such as tuberous sclerosis or von Hippel-Lindau syndrome, should also be considered in the differential diagnosis of renal lymphangiectasia, where in the latter, MRI reveals the cysts at low intensity on T1-weighted images and at high signal intensity on T2-weighted images [12].

Although hydronephrosis is the main differential diagnosis, in contrast to imaging studies, these cystic dilations usually displace the collecting system because of extrinsic compression, and, unlike hydronephrosis, they do not fill with expelled contrast medium [13] as can be seen in the images of our case.

Asymptomatic patients do not require treatment. Conversely, in symptomatic patients, aspiration of the cysts may be an option. The approach of nephrectomy is limited to patients who develop severe symptoms such as high blood pressure and an increase in cysts at the perinephric level or persistent flank pain [14].

As part of the limitations, it is essential to highlight that there are no previous studies that allow us to propose a congenital hypothesis of lymphangiectasia and long-term follow-up that enables us to describe a better evolution of the patient, which is considered essential for a greater understanding of the nature and prognosis of renal lymphangiectasia.

## Conclusions

In conclusion, renal lymphangiectasia is a rare pathology affecting the lymphatic vessels of the kidney, with many symptoms ranging from the absence of clinical manifestations to hematuria and possible systemic repercussions such as arterial hypertension. This case highlights the importance of early detection of renal lymphangiectasia by bedside ultrasound. The fact that this is a benign disease highlights the importance of POCUS for the detection of this rare disease in primary care, as it is easily accessible, time-saving, inexpensive, and minimally invasive for the patient.
